# Simulating self-learning in photorefractive optical reservoir computers

**DOI:** 10.1038/s41598-021-81899-w

**Published:** 2021-01-29

**Authors:** Floris Laporte, Joni Dambre, Peter Bienstman

**Affiliations:** 1 Photonics Research Group, UGent-imec, Technologiepark-Zwijnaarde 126, 9052 Ghent, Belgium; 2 IDLab, UGent-imec, Technologiepark-Zwijnaarde 126, 9052 Ghent, Belgium

**Keywords:** Engineering, Applied optics, Optical materials and structures, Information technology

## Abstract

Photorefractive materials exhibit an interesting plasticity under the influence of an optical field. By extending the finite-difference time-domain method to include the photorefractive effect, we explore how this property can be exploited in the context of neuromorphic computing for telecom applications. By first *priming* the photorefractive material with a random bit stream, the material reorganizes itself to better recognize simple patterns in the stream. We demonstrate this by simulating a typical reservoir computing setup, which gets a significant performance boost on performing the XOR on two consecutive bits in the stream after this initial priming step.

## Introduction

The photorefractive effect can be described as an interesting response of some materials to an applied optical field. When illuminated with light, these materials develop a permanent change in refractive index. The effect, first observed in the 1960s^1,2^, relies on a careful interplay between the photons and the charges in the material which can best be described as follows: photons excite charges in the illuminated regions of the material. These charges are now free to move through the bulk of the material, where they are captured again in the dark (not illuminated) regions of the material. This gives rise to a inhomogeneous charge distribution, which in turn gives rise to a so-called *space-charge* electric field throughout the crystal. Finally, due to the Pockels effect, the varying space charge field has an influence on the refractive index of the material, which in turn will influence the propagation of the light through the crystal.

This interplay between the charge carries in the material and the light propagating through the material make such *photorefractive crystals* ideal candidates for applications involving holography^3,4^ and, as soon as these holographic properties were well understood, these crystals have been used for a variety of applications.

One of these applications, explored in the late 80s and 90s, is neuromorphic computing^5–8^, where the weights of a neural network were written into such a photorefractive crystal in the form of a hologram, yielding a *neural network* with ultra-fast inference. Moreover, it turns out that *optical* training algorithms akin to backpropagation^9^ can be derived for such systems^5^. However, the inherently slow photorefractive process inside the crystal results in quite long training times for such iterative algorithms.

A more viable option for these crystals might however be to integrate them into *optical reservoir computing* setups. Reservoir Computing (RC) is a two-decade old machine learning paradigm^10,11^ used to process time-dependent signals. In RC, a highly dynamical system, the reservoir, is used as a randomized preprocessor to a time-dependent input signal. Preprocessing the input signal this way produces a high-dimensional reservoir state which is subsequently *interpreted* by a simple linear classifier, called the *readout*. The beauty of RC lies in its simplicity: *the same* reservoir is often used for a large number of different applications, while each time only a different readout must be found.

Due to its architectural simplicity, the reservoir computer has found its way into many different optical hardware implementations. Many of which follow the *single-node* reservoir architecture^12–17^, while others follow the *passive* photonic reservoir computing approach^18–21^, which is best characterized by the following equations:

1 $$\begin{aligned} \vec {x}(t)&= W_{\mathrm{in}} \vec {u}(t) + W_{\mathrm{res}} \vec {x}(t-dt) \end{aligned}$$

2 $$\begin{aligned} \vec {y}(t)&= W_{\mathrm{out}} f(\vec {x}(t)). \end{aligned}$$

These two equations describe how the classification of the readout $\vec {y}(t)$ at each time *t* is related to the internal reservoir state $\vec {x}(t)$ through a nonlinear detection operation *f* (which can simply be the quadratic response of a photodiode) and a set of readout weights $W_{\mathrm{out}}$. Moreover, the internal reservoir states $\vec {x}(t)$ at the current timestep are related to the input $\vec {u}(t)$ and reservoir states at the previous timestep $\vec {x}(t-dt)$ through completely passive mixing (without any nonlinearities), characterized by $W_{\mathrm{res}}$.

Traditionally, all the weights are explicitly encoded in software or dedicated hardware. However, the beauty of RC in physical hardware is that $W_{\mathrm{in}}$ and $W_{\mathrm{res}}$ can be completely encoded by a physical system, which allows us to only find a suitable readout $W_{\mathrm{out}}$. In this case $W_{\mathrm{in}}$ and $W_{\mathrm{res}}$ are usually unknown parameters of the physical system.

In this work, we will explore how the *photorefractive effect* can be used to potentially improve such a simple passive optical reservoir computing setup by influencing the internal (unknown) reservoir state $W_{\mathrm{res}}$.

Indeed, a potentially interesting aspect of these photorefractive crystals, is that photorefractive crystals might exhibit a form of self-learning, i.e. the ability to reconfigure themselves according to prolonged exposure to the signals the reservoir system is supposed to classify. It might be possible to exploit this in a way similar to Hebbian learning^22,23^, which is best characterized by the catchphrase *“neurons that fire together wire together”*. In the case of photorefractive crystals in a RC setup, this would take the form of common patterns and correlations in the training input becoming more expressed in the crystal in such a way to make the final classification by the readout easier.

## Results

**Figure 1:**
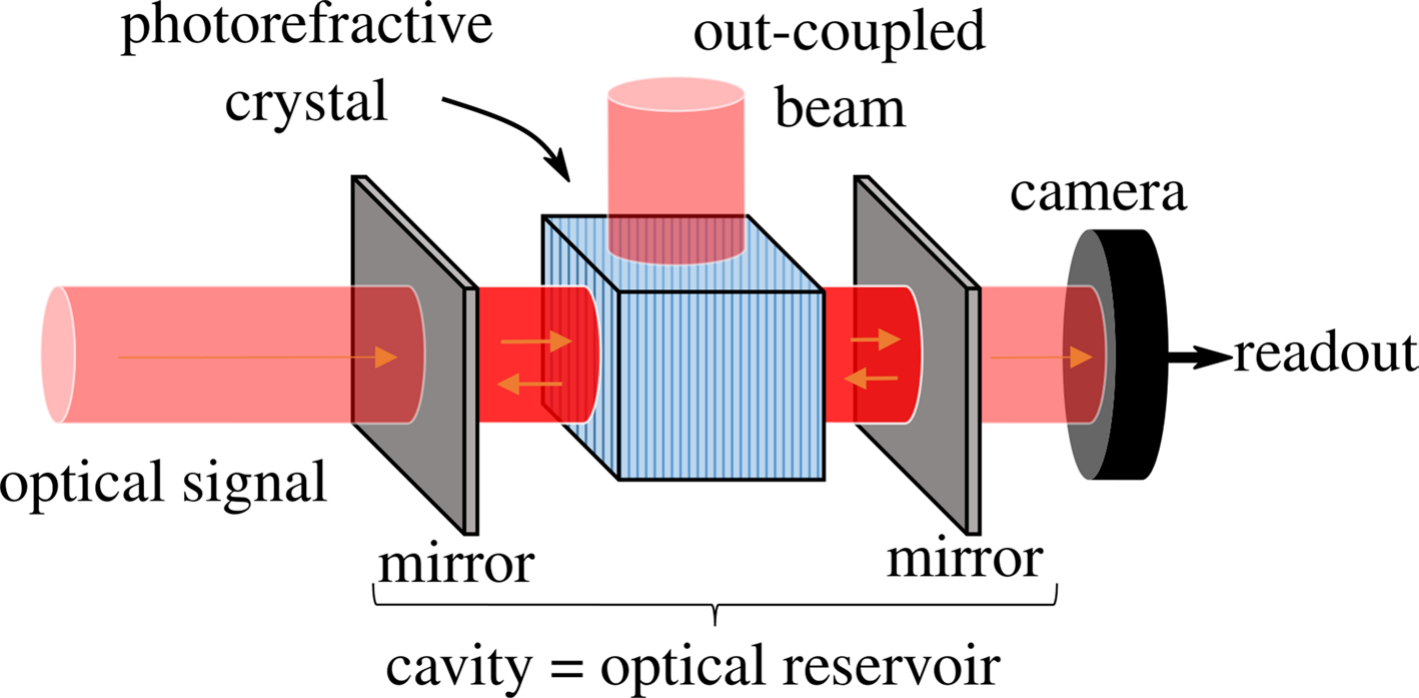
A photorefractive crystal placed in a cavity. Light entering the cavity will interact with the photorefractive crystal such that part of the light will be coupled out of the cavity. Moreover, frequently occurring recurring patterns in the time-dependent input signal will interfere similarly in the crystal resulting in a form of self-learning. This figure was created with Inkscape.

A photorefractive crystal is placed inside a free-space cavity with 50/50 mirrors, as illustrated in Fig. 1. This combination of crystal and cavity will act as the reservoir. Light leaking out of the cavity will be detected by a camera with a limited number of pixels. On these detected camera pixels a readout can be trained for the application at hand.

Within the proposed setup, the photorefractive crystal thus acts as a diffractive element inside the cavity, which introduces the random mixing necessary for a reservoir to function. However, even though this setup resembles typical diffractive optical reservoirs^24–27^, it is important to note that the proposed setup does not contain any nonlinear elements and hence follows the *passive* photonic reservoir setup as introduced in Eq. (1). Indeed, the nonlinear photorefractive effect, which typically acts on a timescale of seconds is typically too slow to have any effect during inference and hence, any charge distribution (and resulting index contrast) within the crystal can be considered constant during inference.

**Figure 2:**
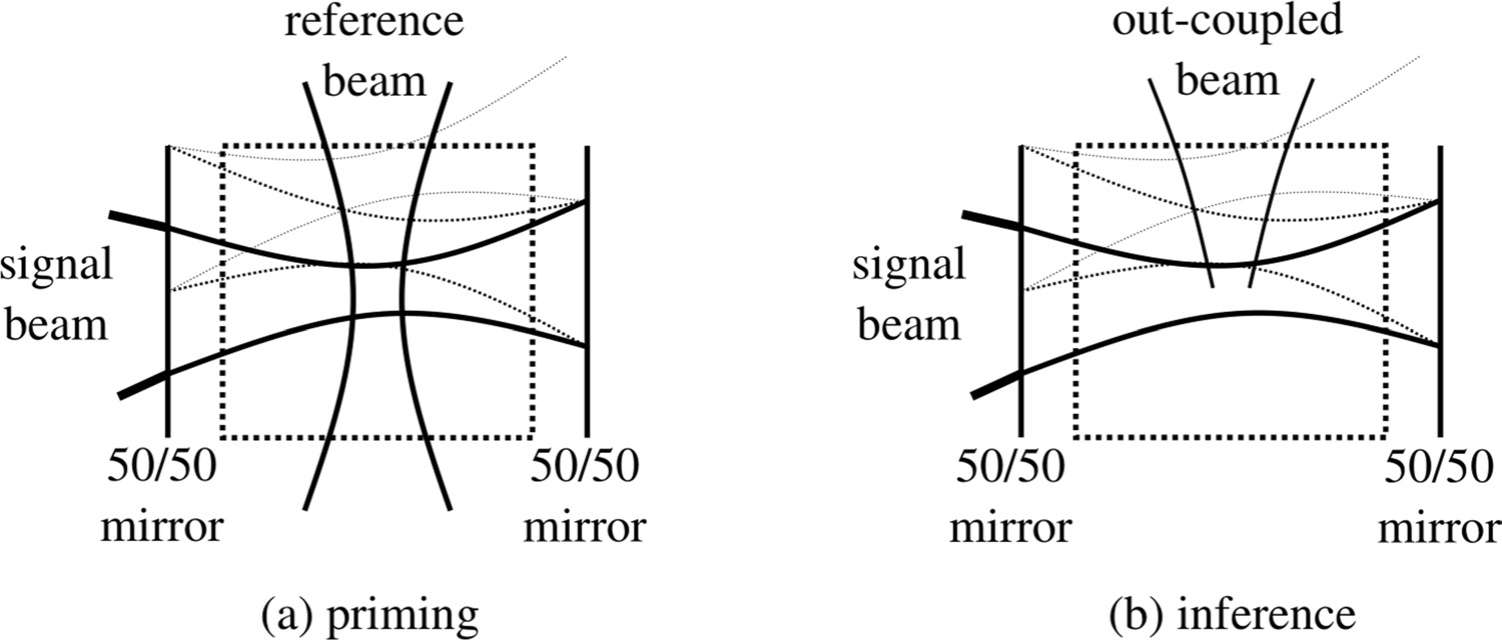
**(a)** During *priming*, a random input signal is sent through the cavity containing a photorefractive crystal while a reference beam is active. The signal beam enters the cavity through a 50/50 mirror and will reflect at the other side of the cavity on another 50/50 mirror. Due to interference with the reference beam (and with reflections of the signal beam itself), induced gratings start to form inside the photorefractive crystal for common patterns in the signal beam. These gratings will in turn influence the propagation of the light through the crystal. When the induced gratings become strong enough, beam coupling occurs. **(b)** During inference, the signal beam will still interact with the gratings created during the priming step These interactions with the photorefractive crystal might give rise to beams that are coupled out of the photorefractive crystal and slower propagation times through the crystal. Overall, such a primed crystal will perform better as a reservoir on the benchmark tasks considered here than the non-primed crystal. This figure was created with Inkscape.

However, whereas traditional reservoir computing setups do not allow any optimization of the reservoir itself, our simulation setup is designed in a way to exploit the self-reorganization of the photorefractive crystal by prolonged exposure to a random bitstream and a reference beam. We will call this initialization procedure the *priming* of the crystal. During priming, a reference beam is active to induce *beam coupling*^28^ inside the crystal between the signal beam and the reference beam, as illustrated in Fig. 2a. The technique of beam coupling in photorefractive crystals is a well-known concept in photorefractive holography^3,4^ and will result in a refractive index distribution that will allow part of the light to be coupled out of the cavity, as illustrated in Fig. 2b during *inference*, after this initial priming step, when the reference beam is turned off.

Generally speaking, a bit entering the cavity will make at least one roundtrip before its amplitude drops below the noise due to power loss at the 50/50 mirrors, losses in the crystal and leakage out of the cavity. These round trips allow for the time-dependent signal to interfere with itself *inside* the crystal. Hence, when the reference beam is active, recurring patterns in the time-dependent input signal will start to couple with the reference beam, resulting in an emerging input signal-dependent index contrast in the crystal.

During inference, the actual bit stream is sent through. The aim is that the initial priming step will have improved the performance of the reservoir setup on the task at hand due to the more pronounced correlations between common bit patterns *inside* the photorefractive crystal. Indeed, even when a purely random bit stream is used for priming, each subsequence of bits (take for example the two-bit sequences 00, 01, 10, 11) will still interact differently with the photorefractive crystal by exciting it slightly differently. This means that those substrings of bits will interact differently with the crystal during inference in a predictable way, which can be classified by the readout.

In our simulations, a bit stream of 10,000 bits is sent through the cavity. When the signal leaks out of the cavity (either behind the mirrors or on the sides of the crystal), the signal will be detected by a camera, consisting of 64 recorded pixel values sampled eight times per bit, which are obtained by spatial averaging of the FDTD grid at the camera location and by performing a lowpass filtering with a cutoff frequency equal to the bitrate for each of the pixels. The readout weights are then trained to follow a boolean target function. Two target functions are considered: a simple copy task, where the same output should be reproduced with a certain latency and the XOR task, where the XOR of two consecutive bits in a bitstream is performed by the system and which also must be reproduced with a certain latency. Training of the readout is, like usually in RC, simply done by linear regression: the chosen readout minimizes the mean squared error between target and prediction. Moreover, after performing a threshold on the predicted output, the Bit Error Rate (BER) can be calculated.

As we are targeting boolean tasks, we would like two consecutive bits in a bit stream to be able to interfere inside the crystal. Hence we choose the width of the cavity such that the propagation time between the two mirrors equals the length of a single bit. However, using typical photorefractive parameters for LiNbO$_3$^29–31^ summarized in Table 1 and a target bitrate of $100\,\mathrm{Gbps}$, this would result in a cavity width of about $700\,\upmu$ m. Doing an FDTD simulation for such cavity for a meaningful amount of bits at a wavelength of $1550\,\mathrm{nm}$ is however near impossible. Therefore, for computational reasons, the simulated cavity is made 100 times smaller to $7 \upmu$ m. To compensate for this reduced size, the bitrate in simulation is increased by the same factor to $10\,\mathrm{Tbps}$. Moreover, as the diffractive power of a grating is in general proportional to both the length of the grating *and* its refractive index contrast, the shorter length of the crystal is compensated by an equal and opposite increase of its Pockels coefficients.

Three different cases will be considered: the *primed* crystal as discussed earlier, an empty cavity and a cavity with a crystal with a random diffraction pattern within. The random diffraction pattern is obtained by performing a band-pass filter on white noise within the spatial frequency range of typical gratings inside a photorefractive crystal, i.e. spatial frequencies corresponding to gratings with pitch between $\lambda /2$ (co- or counter propagating beams) and $\lambda /(2\sqrt{2})$ (perpendicular beams) where allowed. Moreover, the standard deviation on this random index contrast was chosen to equal the standard deviation of the grating in the primed case. These two extra cavity setups should offer a fair comparison between the self-learning *priming* approach and more typical random diffraction reservoirs.

**Table 1:** Simulation parameters for the LiNbO$_3$ crystal simulated with FDTD. Each of the actual values^29–31^ is followed by the values used in simulation. The values used in simulation are chosen to partly compensate for the reduced cavity size that can be simulated with an FDTD simulator.

	Parameter	Actual	Simulation	Unit
$L$	Cavity length	$7\cdot 10^{-3}$	$7\cdot 10^{-5}$	${\mathrm{m}}$
$B$	Bitrate	$100$	$10000$	${\mathrm{Gbps}}$
$s$	Photo-ionization cross section	$0.0025$	$0.0025$	${\mathrm{m}}^2/{\mathrm{J}}$
$\beta$	Thermal excitation rate (300K)	$1.0$	$1.0$	${\mathrm{s}}^{-1}$
$\gamma$	Recombination rate	$10^{-15}$	$10^{-15}$	${\mathrm{m}}^3/{\mathrm{s}}$
$\mu$	Mobility	$0.0015$	$0.0015$	${\mathrm{m}}^2/{\mathrm{Vs}}$
$N_D$	Donor density	$6.6\cdot 10^{24}$	$6.6\cdot 10^{24}$	${\mathrm{m}}^{-3}$
$N_D^+$	Initial excited donor density	$3.3\cdot 10^{24}$	$3.3\cdot 10^{24}$	${\mathrm{m}}^{-3}$
$n$	Initial free electron density	$1\cdot 10^{17}$	$1\cdot 10^{17}$	${\mathrm{m}}^{-3}$
$\epsilon$	Static relative permittivity	$32$	$32$	$1$
$\epsilon$	Relative permittivity @ 1500 nm	$4.9$ - $4.6$	$4.9$ - $4.6$	$1$
$r_{22}$	Pockels coefficient	$7$	$700$	${\mathrm{pm}}/{\mathrm{V}}$
$r_{13}$	Pockels coefficient	$10$	$1000$	${\mathrm{pm}}/{\mathrm{V}}$
$r_{33}$	Pockels coefficient	$32$	$3200$	${\mathrm{pm}}/{\mathrm{V}}$
$r_{42}$	Pockels coefficient	$32$	$3200$	${\mathrm{pm}}/{\mathrm{V}}$

### Copy Task

The copy task consists of sending a bit stream through the reservoir and trying to retrieve the same bit stream with a certain delay. Even though no special calculations need to be performed to do this operation, the copy task still serves as the prime measure for the *memory* of the reservoir.

**Figure 3:**
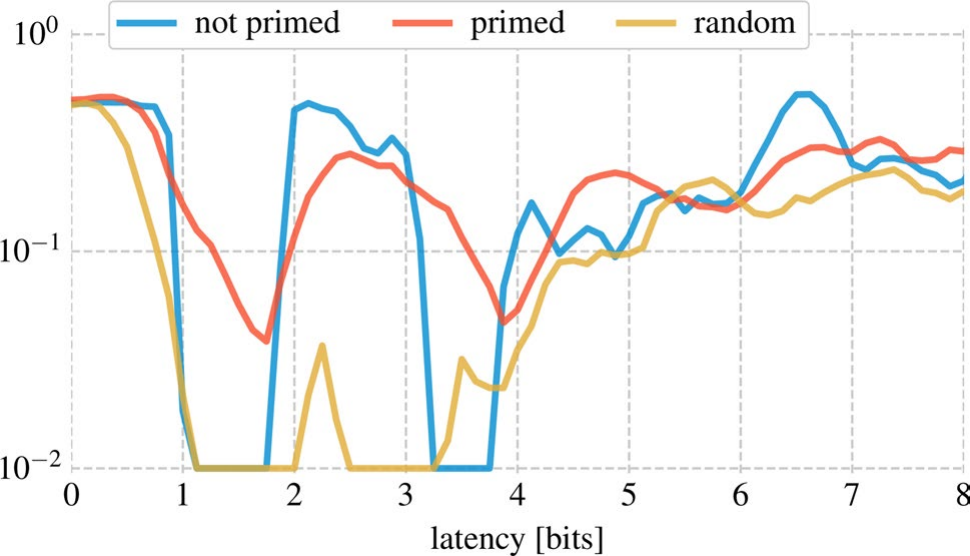
Performance on the copy task before and after priming. Priming has a detrimental effect on the memory of the reservoir. The latency is increased in steps of 0.125 bits.

We attempt to retrieve the original bit stream at different delays or *latencies* after which they were sent out. This is done for a randomly initialized non-primed crystal and a crystal *primed* by the previously described initialization procedure.

In Fig. 3, the latency is increased in steps of 0.125 bits. These steps correspond to the sampling rate of the signal (which is 8 times the bit rate). For each of these latencies, the BER is calculated. Note that latency 0 is defined as when the bit *starts entering* the cavity. As can clearly be seen on the figure, the performance on the copy task degrades after priming, from 0 bits in 10, 000 simulated bits in the non-primed and random case (which corresponds to a *max* BER of $10^{-2}$ for the amount of 10, 000 bits used^32^) to about 200 in 10, 000 in the primed case.

### XOR task

When performing the XOR task, the system is asked to produce the XOR of two consecutive bits in the bitstream. The XOR is a typical benchmark problem in machine learning, as the nonlinearity of the XOR operation makes solving this task non-trivial because the output cannot be found by just performing a linear classification algorithm on the inputs. However, if the mixing in the reservoir is sufficiently large, the non-linearity of the detector is often enough to perform this task^19^. Hence, being able to perform the XOR task in an optical reservoir where a readout is trained on the *detected* reservoir output is often a good indication of sufficient mixing in the reservoir.

**Figure 4:**
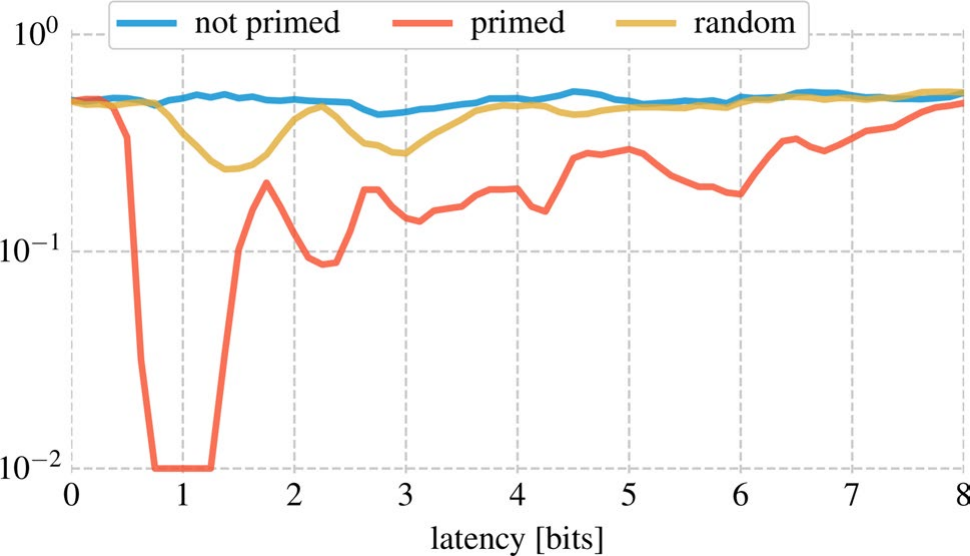
Performance on the XOR task before and after priming. Priming has a beneficial effect on the computational performance of the reservoir. The latency is increased in steps of 0.125 bits.

In Fig. 4, we compare again the performance of the primed reservoir with the non-primed and random reservoir for different latencies (in the case of the XOR, the latency is counted from the moment the *last* bit has *started entering* the cavity), and here we see a stark difference: whereas the primed reservoir is able to perform the XOR between two consecutive bits in the bitstream with 0 errors out of 10, 000 bits, the non-primed reservoir and the random reservoir are totally unable to do so. This might indicate that *priming* the reservoir increases the performance of the reservoir system by trading of some of the memory for computational performance.

## Discussion

This study presents an initial attempt at using *photorefractive materials* for self-learning neuromorphic computing applications. We show that by exposing a photorefractive material to a long, repeated bit stream (priming), the induced gratings make it perform better on a nonlinear time-dependent telecom related benchmark task: the XOR task.

Indeed, performance on the XOR task can be improved from $50\,\%$ BER (random guessing) in the non-primed and random case to 0 errors in 10, 000 simulated bits in the primed case. Moreover, comparing the random grating with similar properties (average index variation and grating pitch) as the primed grating shows much worse performance on that same task, showing that the primed crystal has indeed *learned* to perform the XOR by itself.

However, this gain in computational power comes at the cost of reducing the memory of the reservoir system: tasks requiring more memory (but less computational power) will perform worse. This is exemplified by the results on the copy task, where the primed crystal is unable to copy the bits without any errors.

To perform these self-learning reservoir simulations in a reasonable amount of time, some approximations were necessary. The most important limitation on the simulation was the size of the crystal, which was reduced by a factor 100. To compensate for this smaller crystal size, the bitrate was increased by the same factor, from $100\,\mathrm{Gbps}$ (which would be the target bitrate in an actual physical setup) to $10\,\mathrm{Tbps}$ in simulation. As - generally speaking - the refractive power of a grating is proportional through the index contrast and to its length, we increased the Pockels coefficients in the simulation as well, to compensate for the shorter propagation length through the crystal. All these approximations indicate that the results obtained yield a *qualitative* indication that the proposed system could work in principle on physical hardware. However, actual experimental results are necessary to confirm this claim.

## Methods

### The FDTD method

The Finite Difference Time Domain (FDTD) method^33,34^ is one of the most-used ways to simulate electromagnetic phenomena.

**Figure 5:**
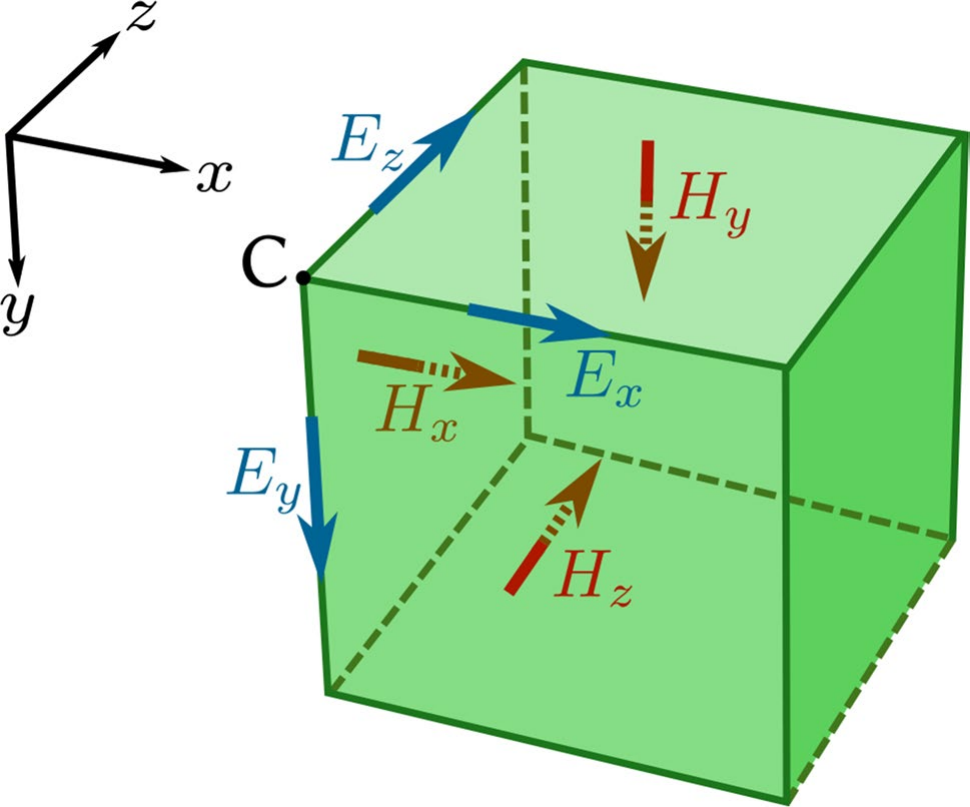
The Yee cell is the grid unit for FDTD simulations. The electromagnetic fields are staggered with half-integer offsets from the corner of the grid cell (marked as *C*). The *E*-fields are on the edges of the unit cell, the *H*-fields are on the faces of the unit cell. This figure was created with Inkscape.

By discretizing the electric field $\vec {E}$ and the magnetic field $\vec {H}$ on a Yee cell, as illustrated in Fig. 5, one can derive the following update equations:

3 $$\begin{aligned} \vec {H}[m,n,p,q+1]&= {\vec {H}}[m,n,p,q] - s_c\mu ^{-1} \vec {\Phi }_E[m,n,p,q] \end{aligned}$$

4 $$\begin{aligned} \vec {E}[m,n,p,q+1]&= \vec {E}[m,n,p,q] + s_c\epsilon ^{-1} \vec {\Phi }_H[m,n,p,q+1], \end{aligned}$$

with

5 $$\begin{aligned} \vec {\Phi }_{E}[m,n,p]&:= \begin{pmatrix} \left( E_z[m,n+1,p]-E_z[m,n,p]\right) - \left( E_y[m,n,p+1]-E_y[m,n,p]\right) \\ \left( E_x[m,n,p+1]-E_x[m,n,p]\right) - \left( E_z[m+1,n,p]-E_z[m,n,p]\right) \\ \left( E_y[m+1,n,p]-E_y[m,n,p]\right) - \left( E_x[m,n+1,p]-E_x[m,n,p]\right) \end{pmatrix} \end{aligned}$$

6 $$\begin{aligned} \vec {\Phi }_{H}[m,n,p]&:= \begin{pmatrix} \left( H_z[m,n,p]-H_z[m,n-1,p]\right) - \left( H_y[m,n,p]-H_y[m,n,p-1]\right) \\ \left( H_x[m,n,p]-H_x[m,n,p-1]\right) - \left( H_z[m,n,p]-H_z[m-1,n,p]\right) \\ \left( H_y[m,n,p]-H_y[m-1,n,p]\right) - \left( H_x[m,n,p]-H_x[m,n-1,p]\right) \end{pmatrix} \end{aligned}$$

Where *q* represents the time-index of the simulation and the indices *m*, *n*, *p* represent the index of the Yee-cell the field components belong to along the *x*, *y* and *z* axis respectively (half-integer offsets from the corner of the grid-cell as laid out in Fig. 5 are implicitly assumed). Moreover, $s_c$ is known as the Courant number of the simulation, which—for a 3D simulation—must satisfy the following stability requirement.

7 $$\begin{aligned} s_c=\frac{cdt}{du} \le \frac{1}{\sqrt{3}} \end{aligned}$$

with *dt* the timestep of the simulation and *du* the grid spacing.

### Kukhtarev equations

Numerically modeling the photorefractive effect comes down to integrating the Kukhtarev equations^35^ into the FDTD method. These equations

8 $$\begin{aligned} \frac{dn}{dt}&= \left. \frac{dn}{dt}\right| _{N_D} + \left. \frac{dn}{dt}\right| _{J} = \frac{dN_D^+}{dt}+\nabla \cdot \vec {J} \end{aligned}$$

9 $$\begin{aligned} \frac{dN_D^+}{dt}&= (sI+\beta )(N_D-N_D^+)-\gamma n N_D^+ \end{aligned}$$

10 $$\begin{aligned} \vec {J}&= \frac{\mu k T}{e}\nabla n - \mu n \vec {S} \end{aligned}$$

describe respectively how the change in free electron density *n* in the photorefractive material (Eq. (8)) is related to two processes. The first process (Eq. (9)) describes the excitation of the free carriers *n* from neutral donors $N_D$ (related to the intensity *I* of the incident optical field, the photo-ionization constant *s* and the thermal excitation constant $\beta$) and recombination with positively charged traps $N_D^+$ (according to a recombination constant $\gamma$). The second process (Eq. (10)) describes the diffusion of the free carriers through the material due to the non-uniform charge distribution $\nabla n$ and its resulting space-charge field $\vec {S}$, where $\mu$ represents the mobility of the free carriers, *e* represents the elementary charge and *k* is the Boltzmann constant.

The equations, as they are described here, assume no difference between traps and donors in the photorefractive material: each unfilled trap is positively charged and conversely each filled trap is a (neutral) donor. Second, it is implicitly assumed that each trap has the same excitation energy and the excitation energy needed to excite electrons from the valence band is too high to have any influence.

### Generation and recombination

In Eq. (9), the change in excited donor density $N_D^+$ can be split into a *generative* term and a *recombination* term. The generative term will be proportional (through a photo-ionization cross-section *s*) to the intensity of the light *I*, which in this case is defined in terms of the energy density $I=c{\mathcal {E}}$. Assuming the only absorption in the photorefractive material is due to the photo-ionization, we can propose a relation between the *photo-ionization* *s* and the absorption coefficient in the material $\alpha$:

11 $$\begin{aligned} \alpha&= s \frac{h c}{\lambda } (N_D - N_D^+) \end{aligned}$$

Note that the assumption that the absorption is completely due to the photo-ionization is an approximation. It gives a lower bound for the absorption, given the photo-ionization cross-section *s*. Moreover, free carriers will also be uniformly generated due to a thermal excitation rate $\beta$. On the other hand, the recombination term will be proportional to the number of free electrons *n* and the number of excited donors $N_D^+$ through a recombination rate $\gamma$.

### Electron diffusion

The carrier density will also be influenced by diffusion, which is related to Eq. (10) in the following way:

12 $$\begin{aligned} \left. \frac{\partial n}{\partial t}\right| _{J} = \nabla \cdot J&= \left. \frac{\partial n}{\partial t}\right| _{\mathrm{diff}} + \left. \frac{\partial n}{\partial t}\right| _{\mathrm{drift}} \end{aligned}$$

13 $$\begin{aligned}&= D\nabla ^2 n - \nabla \cdot \vec {F} \end{aligned}$$

Here, we defined the diffusion constant $D=\mu kT/e$ and the electron flow $\vec {F}= n \mu \vec {E}$. This diffusion equation can be discretized on the Yee-grid with symmetric differences (*n* is chosen to be on the *corners* of the Yee-cell):

14 $$\begin{aligned} n'[{m,n,p}] =&n[{m,n,p}] + \frac{Ddt}{du^2} \big (n[m+1,n,p]+n[m-1,n,p]\big . + n[m,n+1,p]+n[m,n-1,p]\nonumber \\&\big . ~ +n[m,n,p+1]+n[m,n,p-1]-6n[m,n,p] \big ) - \frac{dt}{2du} \big ({F_x[{m+1,n,p}]-F_x[{m-1,n,p}]}\big . \nonumber \\&\big . ~ + F_y[{m,n+1,p}]-F_y[{m,n-1,p}] + F_y[{m,n,p+1}]-F_y[{m,n,p-1}]\big ) \end{aligned}$$

However, by carefully fixing the time step for this update equation to be

15 $$\begin{aligned} dt&= \frac{du^2}{6D} = \frac{e du^2}{6kT\mu }, \end{aligned}$$

the update equation gets considerably simplified:

16 $$\begin{aligned} n'[{m,n,p}] =&\frac{1}{6}\big ( {n[{m+1,n,p}]+n[{m-1,n,p}]}+{n[{m,n+1,p}]+n[{m,n-1,p}]} \big .\nonumber \\&~+n[m,n,p+1]+n[m,n,p-1]\big )- \frac{edu}{12kT\mu }\big ({F_x[{m+1,n,p}]-F_x[{m-1,n,p}]}\big . \nonumber \\&~+ {F_y[{m,n+1,p}]-F_y[{m,n-1,p}]}+ F_y[{m,n,p+1}]-F_y[{m,n,p-1}]\big ) \end{aligned}$$

This equation would reduce to the typical Lax–Friedrich scheme if the space-charge field were to be uniform.

### Space charge electric field

The diffusion of the free carriers depends on the space-charge field *S* through Eq. (16). However, the space-charge field itself is related to the free carrier distribution *n* through the charge density $\rho =e(N_D^+-n)$:

17 $$\begin{aligned} \nabla \cdot \vec {S}&= \frac{\rho }{\epsilon _s} \end{aligned}$$

18 $$\begin{aligned} \nabla \times \vec {S}&= \vec {0} \end{aligned}$$

Here, $\epsilon _s$ is the *static* permittivity of the photorefractive material, which usually is vastly different than the permittivity at optical wavelengths. Moreover, it is also assumed that *S* varies slowly enough to allow the second equation to equal zero.

The space-charge field is an electric field and hence lives on the edges of the Yee cell. Moreover, *n* is located on the corners of the Yee-cell, hence Eq. (17) can be discretized as follows:

19 $$\begin{aligned} S_x[m,n,p] - S_x[m-1,n,p] + S_y[m,n,p] - S_y[m,n-1,p]&\nonumber \\ ~+ S_z[m,n,p] - S_z[m,n,p-1]&= \frac{\rho [m,n,p]}{\epsilon _s} \end{aligned}$$

Moreover, the application of the curl (Eq. 18) needs to be solved on the faces of the grid cell:

20 $$\begin{aligned} S_z[m,n+1,p] - S_z[m,n,p] - S_y[m,n,p+1] + S_y[m,n,p]=0 \end{aligned}$$

21 $$\begin{aligned} S_x[m,n,p+1] - S_x[m,n,p] - S_z[m+1,n,p] + S_z[m,n,p]=0 \end{aligned}$$

22 $$\begin{aligned} S_y[m+1,n,p] - S_y[m,n,p] - S_x[m,n+1,p] + S_x[m,n,p]=0 \end{aligned}$$

Taking all the equations together for each grid point gives the overdetermined system

23 $$\begin{aligned} A\vec {x}&= \vec {b}. \end{aligned}$$

Where $\vec {x}$ is the vector of 3*MNP* unknowns and $\vec {b}$ the vector of 4*MNP* targets:

24 $$\begin{aligned} \vec {x}&= \left( S_x[1,1,1], \cdots , S_x[M,N,P], S_y[1,1,1], \cdots , S_z[M,N,P] \right) ^T \end{aligned}$$

25 $$\begin{aligned} \vec {b}&= \left( \rho [0,0,0]/\epsilon _s, \cdots , \rho [M,N,P]/\epsilon _s, 0, \cdots , 0 \right) ^T. \end{aligned}$$

Moreover, $A$ is a sparse matrix containing the coefficients of Eqs. (19) and (22). Although this system overdetermined, it turns out a solution to this linear system of equations can still be found by using the left pseudo-inverse of *A*:

26 $$\begin{aligned} \vec {x} = (A^TA)^{-1}A^T \vec {b} \end{aligned}$$

We use the biconjugate gradient method^36^ to solve this system every diffusion timestep. The biconjugate gradient method is efficient because it solves for $A^TA$ iteratively and hence no inversion of a sparse matrix (which is generally speaking not sparse itself) is necessary. Moreover, the biconjugate gradient method also allows to initialize the system with an estimate of *x* for which the value of *x* at the previous diffusion time step can be used.

### The electro-optic effect

Finally, a relation between this space charge electric field $\vec {S}$ and the optical properties of the material still needs to be found. Generally speaking, the electro-optic effect is described as a dependence of the *impermeability* tensor $\eta =\mu _r\epsilon _r^{-1}$ of the material on a present electric field $\vec {E}$. When this electric field is small—as is the case for the space charge electric field $\vec {S}$—a first-order series expansion can be used:

27 $$\begin{aligned} \eta _{ij}(\vec {S})&= \eta _{ij}(0) + r_{ijk} S_k. \end{aligned}$$

This first-order dependency on the electric field is called the Pockels effect. In the case of non-magnetic materials ($\mu _r = 1$), this equation can be rewritten as:

28 $$\begin{aligned} (\epsilon _{r}^{-1})_{ij}(\vec {S})&= (\epsilon _r^{-1})_{ij}(0) + r_{ijk} S_k. \end{aligned}$$

This equation mixes *E*-type field components, located on the edges of the Yee cell with *H*-type field components, located on the faces of the Yee-cell. This often causes numerical instabilities as it is not clear how to handle the non-diagonal components of $\epsilon _r^{-1}$. To solve this problem, the method proposed in by Werner et al.^37^ can be used, which proposes a modified but stable update equation for the electric fields:

29 $$\begin{aligned} \vec {E}_x&~\texttt {+=}~\begin{bmatrix} \epsilon _r^{-1} \begin{pmatrix} 0 \\ {(\vec {\Phi }_{H})}_y^{\{C\}} \\ {(\vec {\Phi }_{H})}_z^{\{C\}} \end{pmatrix} \end{bmatrix}^{\{E_x\}}_x + {(\epsilon _r^{-1})}^{\{E_x\}} \begin{pmatrix} {(\vec {\Phi }_{H})}_x \\ 0 \\ 0 \end{pmatrix}\nonumber \\ \vec {E}_y&~\texttt {+=}~\begin{bmatrix} \epsilon _r^{-1} \begin{pmatrix} {(\vec {\Phi }_{H})}_x^{\{C\}} \\ 0 \\ {(\vec {\Phi }_{H})}_z^{\{C\}} \end{pmatrix} \end{bmatrix}^{\{E_y\}}_y + {(\epsilon _r^{-1})}^{\{E_y\}} \begin{pmatrix} 0 \\ {(\vec {\Phi }_{H})}_y \\ 0 \end{pmatrix} \nonumber \\ \vec {E}_z&~\texttt {+=}~\begin{bmatrix} \epsilon _r^{-1} \begin{pmatrix} {(\vec {\Phi }_{H})}_x^{\{C\}} \\ {(\vec {\Phi }_{H})}_y^{\{C\}} \\ 0 \end{pmatrix} \end{bmatrix}^{\{E_z\}}_z + {(\epsilon _r^{-1})}^{\{E_z\}} \begin{pmatrix} 0 \\ 0 \\ {(\vec {\Phi }_{H})}_z \end{pmatrix}, \end{aligned}$$

where ${(\cdot )}^{\{C\}}$ is defined as an interpolation of a field component to the corner of the grid cell and ${(\cdot )}^{\{E_i\}}$ is defined as an interpolation of a field component to the $E_i$-edge of the grid cell.

### Towards a photorefractive FDTD simulation

**Figure 6:**
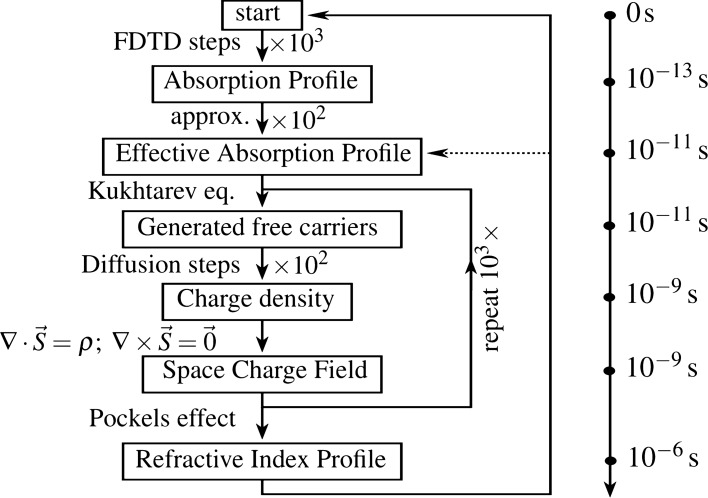
A flowchart of a typical photorefractive FDTD simulation together with typical times in the physical process. Each iterative operation is accompanied by a typical number of iterations. This figure was created with Inkscape.

How all these different photorefractive processes are merged into a modified FDTD simulation is visualized in Fig. 6, which also shows the different timescales each of these processes operate on.

The FDTD timescale is best characterized by its time step, which usually is around $dt_{\mathrm{FDTD}}\approx 0.1\,{\mathrm{fs}}$. Depending on the size of the grid, the FDTD simulation is run for a few thousand time steps. As a general rule we assume it takes about 1000 FDTD time steps to simulate the propagation of a single pulse through the photorefractive material at hand, hence after the FDTD simulation, about $100\,\mathrm fs$ has passed.

However, the diffusion in the crystal happens at a much slower pace. Using (15) we can find that for a typical photorefractive material like LiNbO$_3$, with a mobility $\mu =0.0015\, \mathrm m^2/Vs$^31^, the diffusion time step is about $dt_{\mathrm{diff}} \approx 15\,\mathrm ps$—about 2 orders of magnitude larger than the *full* FDTD simulation. During this characteristic time of the diffusion, the refractive index of the material can be considered constant. To save simulation time, we multiply the absorption profile obtained through the FDTD simulation with a factor 100, which would physically be roughly equivalent to sending the same signal 100 times through the crystal.

The absorption profile can then be converted into free carriers through Eqs. (11) and (9). These are then free to diffuse through the crystal with the mentioned diffusion time step. Typically we will update the space charge field about every 100 diffusion steps and repeat this process 1000 times, which means that the refractive index is updated every $10^{-6}\,\mathrm s$ before the whole simulation process is started over again.

## Data Availability

Simulation files and results can be obtained from the corresponding author upon reasonable request.
